# Efficient biocatalyst by encapsulating lipase into nanoporous gold

**DOI:** 10.1186/1556-276X-8-180

**Published:** 2013-04-19

**Authors:** Xiaoyu Du, Xueying Liu, Yufei Li, Chao Wu, Xia Wang, Ping Xu

**Affiliations:** 1 State Key Laboratory of Microbial Technology, Shandong University, Jinan 250100, People’s Republic of China

**Keywords:** Lipase, Immobilization, Nanoporous gold, Catalysis, Reusability

## Abstract

Lipases are one of the most important biocatalysts for biotechnological applications. Immobilization is an efficient method to increase the stability and reusability of lipases. In this study, nanoporous gold (NPG), a new kind of nanoporous material with tunable porosity and excellent biocompatibility, was employed as an effective support for lipase immobilization. The pore size of NPG and adsorption time played key roles in the construction of lipase-NPG biocomposites. The morphology and composition of NPG before and after lipase loading are verified using a scanning electron microscope, equipped with an energy-dispersive X-ray spectrometer. The resulting lipase-NPG biocomposites exhibited excellent catalytic activity and remarkable reusability. The catalytic activity of the lipase-NPG biocomposite with a pore size of 35 nm had no decrease after ten recycles. Besides, the lipase-NPG biocomposite exhibited high catalytic activity in a broader pH range and higher temperature than that of free lipase. In addition, the leaching of lipase from NPG could be prevented by matching the protein’s diameter and pore size. Thus, the encapsulation of enzymes within NPG is quite useful for establishing new functions and will have wide applications for different chemical processes.

## Background

Immobilization of enzymes on insoluble supports is a significant process due to its promising potential in improving enzyme thermal or pH stability, easing product purification, and facilitating enzyme recycling [1,2]. Therefore, immobilized enzymes have a broader range of applications such as bioconversion, bioremediation, biodetection, and biosensing [3-8].

Among the various supports used for enzyme immobilization, nanoporous gold (NPG) has attracted much attention recently [9-12]. NPG, fabricated by a simple dealloying method, possesses the following unique properties: (1) it is a bulky material with microstructure, which means it can be easily employed and recovered; (2) it has an open three-dimensional structure while possessing a comparably high surface area, which favors strong adsorption and can afford high enzyme loading; (3) the pore size is tunable in a wide range from a few nanometers to many microns, which fits for a wide range of enzyme molecules with specific molecular weight and function; (4) it well reserves a biocompatible, clean, and active surface, which efficiently alleviates enzyme denaturation. Coupled with a rich surface chemistry for further functionalization and excellent conductivity, NPG has great potential for applications in heterogeneous catalysis, electrocatalysis, fuel cell technologies, and biomolecular sensing in comparison with other mesoporous materials [10-13].

In our previous work, enzyme-NPG biocomposites were successfully constructed by assembling various enzymes (such as lipase, catalase, and horseradish peroxidase) onto NPG [12]. Among these enzymes, lipase has gained particular interest as one of the most frequently used biocatalysts in the hydrolysis and the synthesis of esters from glycerol and long-chain fatty acids [14]. In addition, lipase is commercially important and has many applications in food industry and clinical analysis [15]. Especially, lipases are important drug targets or marker enzymes in the medical field. Recently, the development of lipase sensors has been strongly focused on biosensors for the detection of triglycerides and cholesterol [16]. Therefore, further studies were carried out on the catalytic performance of the lipase-NPG biocomposite in this study. It is revealed that the pore size of NPG and adsorption time play significant roles in enzyme loading, leaching, activity, and reusability. The finding should be useful for the creation of biocatalysts and biosensors.

## Methods

4-Nitrophenyl palmitate, *p*-nitrophenol, pyrogallol, and lipase (Aldrich 534641 from *Pseudomonas cepacia*) were purchased from Sigma-Aldrich (St. Louis, MO, USA).

NPG was made by chemically dealloying AgAu alloy foils (Ag_78_Au_22_ at.%, 25 μm in thickness, purchased from Changshu Noble Metal Company, China) in concentrated HNO_3_ (approximately 67%). NPG with a pore size of 35 nm was obtained by chemically dealloying AgAu alloy foils in concentrated HNO_3_ (approximately 67%) at 30°C for 2 h. The preparation of NPG with a pore size of 100 nm was that AgAu alloy foils was chemically dealloyed in concentrated HNO_3_ (approximately 67%) at 30°C for 2 h and then annealed at 250°C for 10 min. After rinsing in distilled water, the samples were dried and kept in a desiccator for further use. The morphology of the samples was observed with a JSM-6700 F field emission scanning electron microscope (SEM; JEOL Ltd., Tokyo, Japan), equipped with an Oxford INCA x-sight energy-dispersive X-ray spectrometer (EDS; Oxford Instruments, Abingdon, Oxfordshire, UK) for compositional analysis.

The immobilized lipase was prepared as previously described [12]. For enzyme immobilization, 1 ml of lipase solution (1.0 mg ml^−1^ of lipase in 50 mM, pH 8.0 Tris–HCl buffer) was mixed with 18 mg of NPG. Then, the mixture was incubated at 4°C without shaking for a certain period of time. After incubation, the supernatant was removed by centrifugation (5,000×*g* for 5 min), and the resulting lipase-NPG biocomposite was washed five times with Tris–HCl buffer (50 mM, pH 8.0) to remove the weakly adsorbed enzyme. The amount of immobilized enzyme was determined by Bradford protein assays [17].

For leaching test, the lipase-NPG biocomposite was incubated in Tris–HCl buffer (50 mM, pH 8.0) for 0.5 and 5 h at 40°C, respectively. Then, the Tris–HCl buffer was removed. The catalytic activity of the lipase-NPG biocomposite was determined.

The catalytic activities of free lipase and the lipase-NPG biocomposite were determined by measuring the initial hydrolysis rate of 4-nitrophenyl palmitate (pNPP) by lipase at 40°C, using a spectrophotometer (2100), following the increase of *p*-nitrophenol (pNP) concentration at 410 nm [12]. One unit (U) of catalytic activity is defined as the amount of lipase which catalyzes the production of 1 μg *p*-nitrophenol under the experimental conditions. For reusability test, the lipase-NPG biocomposite was washed with Tris–HCl buffer (50 mM, pH 8.0) for three times after catalytic activity determination in each cycle, and then used in the next cycle.

## Results and discussion

### Characterization of lipase-NPG biocomposites

**Figure 1:**
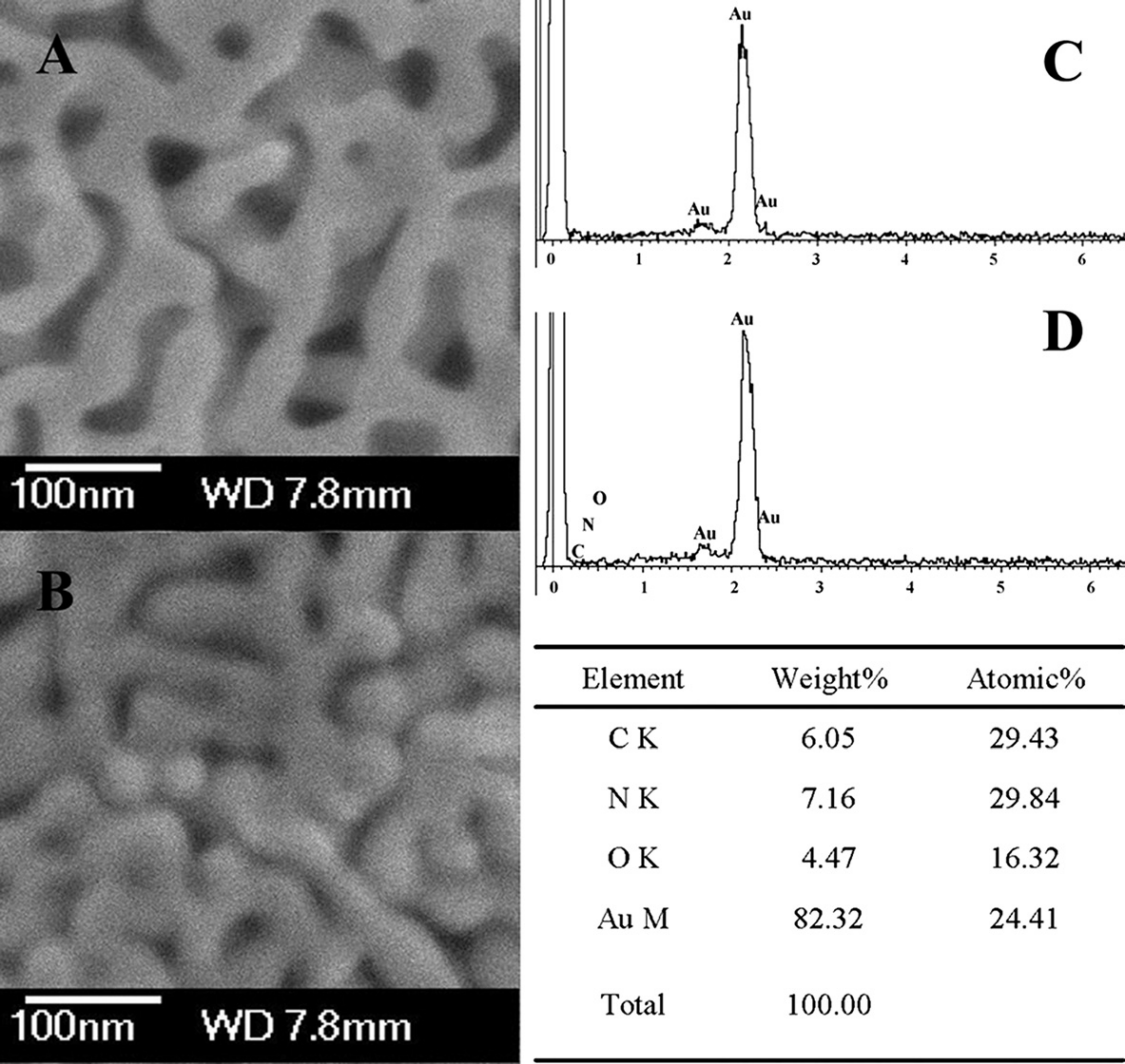
**SEM images of NPG with a pore size of 35 nm.** (**A**) Before and (**B**) after lipase loading, and (**C**, **D**) its corresponding EDS spectra, respectively.

Samples of NPG (pore size of 35 nm) before and after lipase loading were characterized using SEM. Figure 1A illustrates an open three-dimensional nanoporous structure. EDS compositional analysis reveals that only Au was observed, indicating that the residual Ag is below the detection limit of about 0.5% (Figure 1C). After lipase loading, the pores of NPG were filled and the edge of ligaments became dim (Figure 1B) compared with bare NPG (Figure 1A). In addition, EDS analysis confirmed the existence of dominant elements such as C, N, and O (Figure 1D), providing a primary evidence of successful lipase immobilization on NPG.

### Catalytic activity of lipase-NPG biocomposites

**Figure 2:**
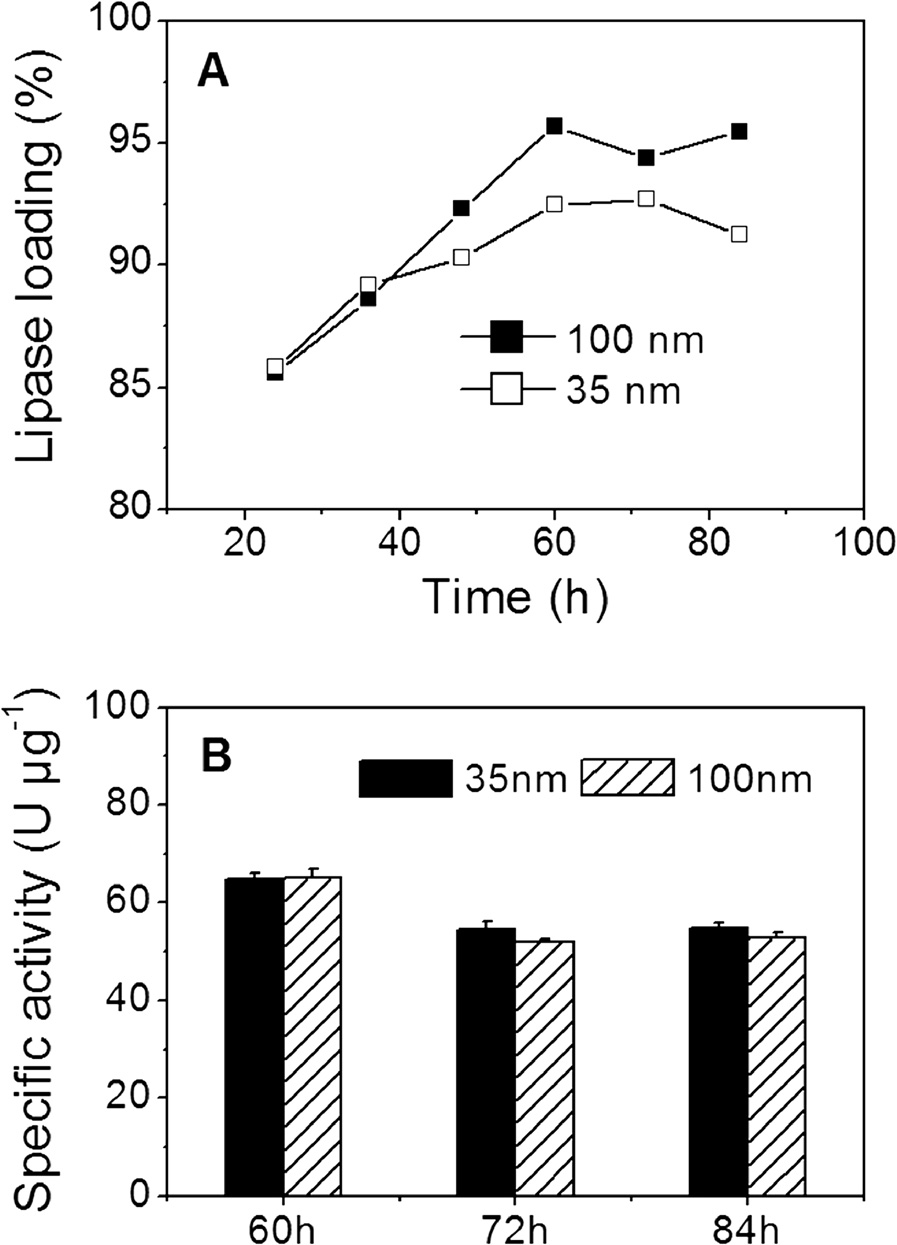
**Lipase loading and catalytic activity.** (**A**) Loadings of lipase and (**B**) catalytic activity of the lipase-NPG biocomposites with pore sizes of 35 and 100 nm.

For the immobilization of lipase, the suitability of NPG with pore sizes of 35 and 100 nm was investigated, respectively. As shown in Figure 2A, similar adsorption profiles were obtained for NPG with pore sizes of 35 and 100 nm. The loadings of lipase on NPG with pore sizes of 35 and 100 nm all reached stationary phase at 60 to 84 h simultaneously. At equilibrium state, the lipase loadings were all higher than 90% of the initial protein amount. The dimension of lipase revealed by its crystal structure as reported is about 5 nm [18]. The pore sizes of NPG (35 and 100 nm) are about 7 and 20 times the dimension of the lipase molecule, respectively. These results indicate that the pore sizes of 35 and 100 nm were large enough to allow lipase to enter the internal pores and porous volume of NPG, which resulted in high lipase loadings. Thus, matching the protein’s diameter and pore diameter is a critical factor in attaining high loading [7].

High enzyme loading alone is not enough to ensure high catalytic activity and stability. As discussed above, high lipase loadings were successfully obtained during the adsorption period from 60 to 84 h. Therefore, the catalytic activity and stability of the lipase-NPG biocomposites were examined after adsorption for 60, 72 and 84 h, respectively. As shown in Figure 2B, the lipase-NPG biocomposite with a pore size of 35 nm had the catalytic activities of 64.8, 54.4 and 54.7 U μg^−1^ protein after adsorption for 60, 72 and 84 h, respectively. On the other hand, the catalytic activities of the lipase-NPG biocomposite with a pore size of 100 nm were 65.1, 52.1 and 52.9 U μg^−1^ protein, respectively. Compared with free lipase (Table 1), no significant decrease on catalytic activity was observed for the lipase-NPG biocomposites with pore sizes of 35 and 100 nm. Additionally, the control experiments show that no decrease was observed on the catalytic activity of free lipase during the adsorption process as shown in Table 1. These results indicate that NPG with pore sizes of 35 and 100 nm not only supported high enzyme loading, but also maintained high catalytic activity compared with other insoluble material systems [19,20]. In contrast, the catalytic activity for *Candida rugosa* lipase immobilized on *γ*-Fe_2_O_3_ magnetic nanoparticles (1.6 × 10^−7^ mol/min per mg of protein) is lower than that for the free enzyme (2.6 × 10^−5^ mol/min per mg of protein) [19]. In addition, the maximal activity recovery of the lipase immobilized on mesoporous silica (average pore diameter 30 nm) was only 76% [20].

**Table 1:** The catalytic activity of free lipase during adsorption processes

	**Adsorption time (h)**			
	**0**	**60**	**72**	**84**
Catalytic activity (U μg^−1^ protein)	55.7 ± 1.7	54.3 ± 2.7	54.8 ± 3.1	57.6 ± 0.9

### Reusability of lipase-NPG biocomposites

**Figure 3:**
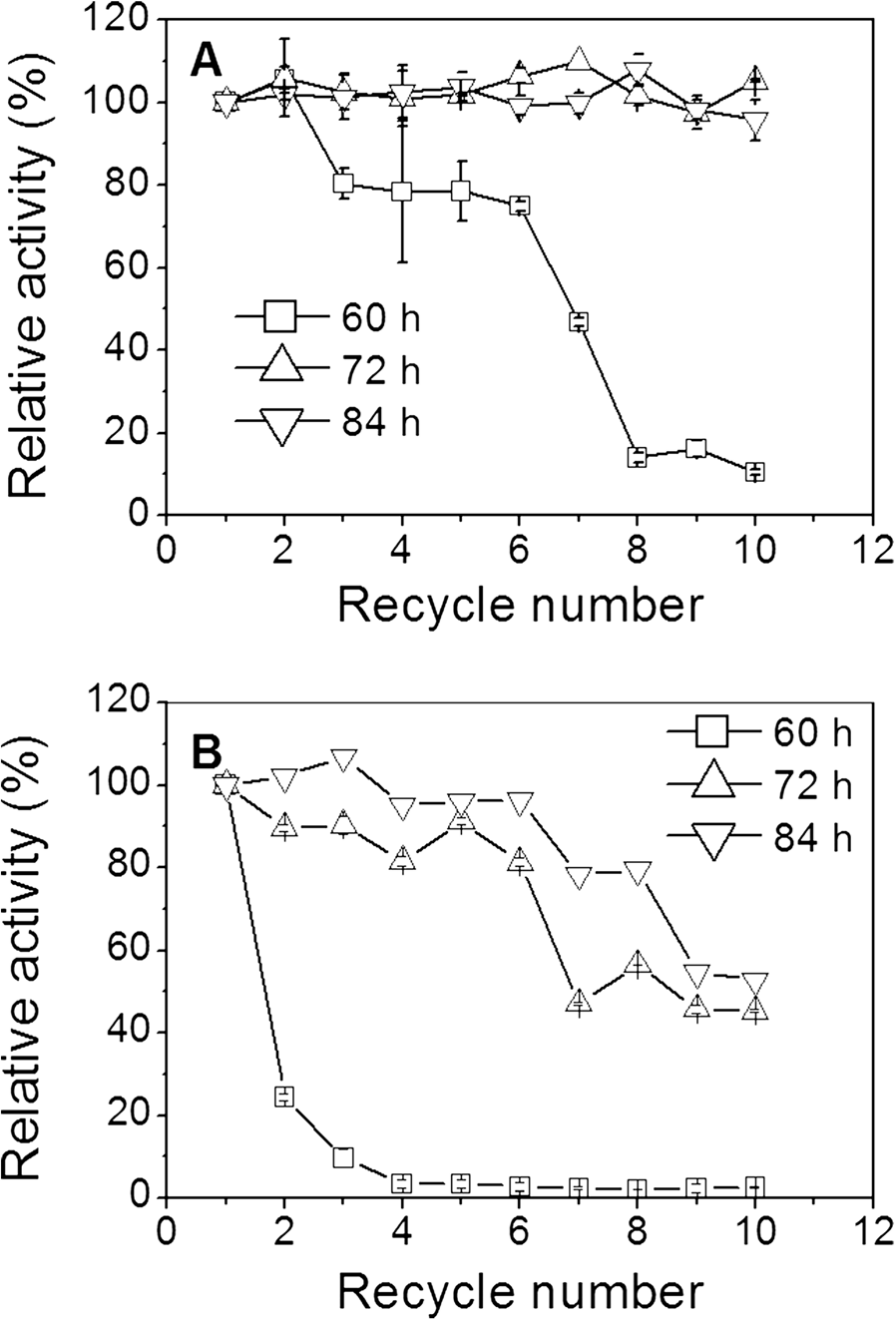
Reusability of lipase-NPG biocomposites with pore sizes of (A) 35 nm and (B) 100 nm.

Reusability is one attractive advantage of immobilized enzymes, which could decrease the cost of enzyme in practical application. The reusability of the lipase-NPG biocomposites was also evaluated. As shown in Figure 3A, when NPG with a pore size of 35 nm served as a support, the lipase-NPG biocomposites adsorbed for 72 and 84 h all exhibited excellent reusability, and no catalytic activity decrease was observed after ten recycles. However, the lipase-NPG biocomposite adsorbed for 60 h exhibited a significant decrease on catalytic activity after six recycles (Figure 3A). The reason may be that most of the lipase is only adsorbed on the external surface of NPG rather than encapsulated within the pores of NPG due to the shorter adsorption time, which resulted in the leaching of lipase from NPG and the poor operational stability for the lipase-NPG biocomposite adsorbed for 60 h upon recycling. In contrast, when NPG with a pore size of 100 nm served as a support, the lipase-NPG biocomposites adsorbed for 60, 72, and 84 h all exhibited significant decreases on catalytic activities during the recycle process (Figure 3B). This may be due to the leaching of lipase from NPG with larger pore size, resulting in the loss of lipase activity upon the reuse process [7]. Based on the above results, it is clear that the pore size of NPG and adsorption time played key roles in achieving high stability and reusability for the lipase-NPG biocomposites. The lipase-NPG biocomposites with a pore size of 35 nm adsorbed for 72 h exhibited excellent reusability and had no decrease on catalytic activity after ten recycles. In comparison, there was 60% of its initial catalytic activity after the fifth cycle by lipase encapsulated in the porous organic–inorganic system [21], and there was 20% of its initial catalytic activity after 7 cycles by lipase immobilized on alginate [22]. The lipase immobilized on surface-modified nanosized magnetite particles showed a significant loss in activity after the first use [23]. Therefore, the lipase-NPG biocomposites with a pore size of 35 nm adsorbed for 72 h was further discussed in the subsequent experiments due to high lipase loading and excellent catalytic performance.

### Effect of buffer pH and temperature on lipase-NPG biocomposite

**Figure 4:**
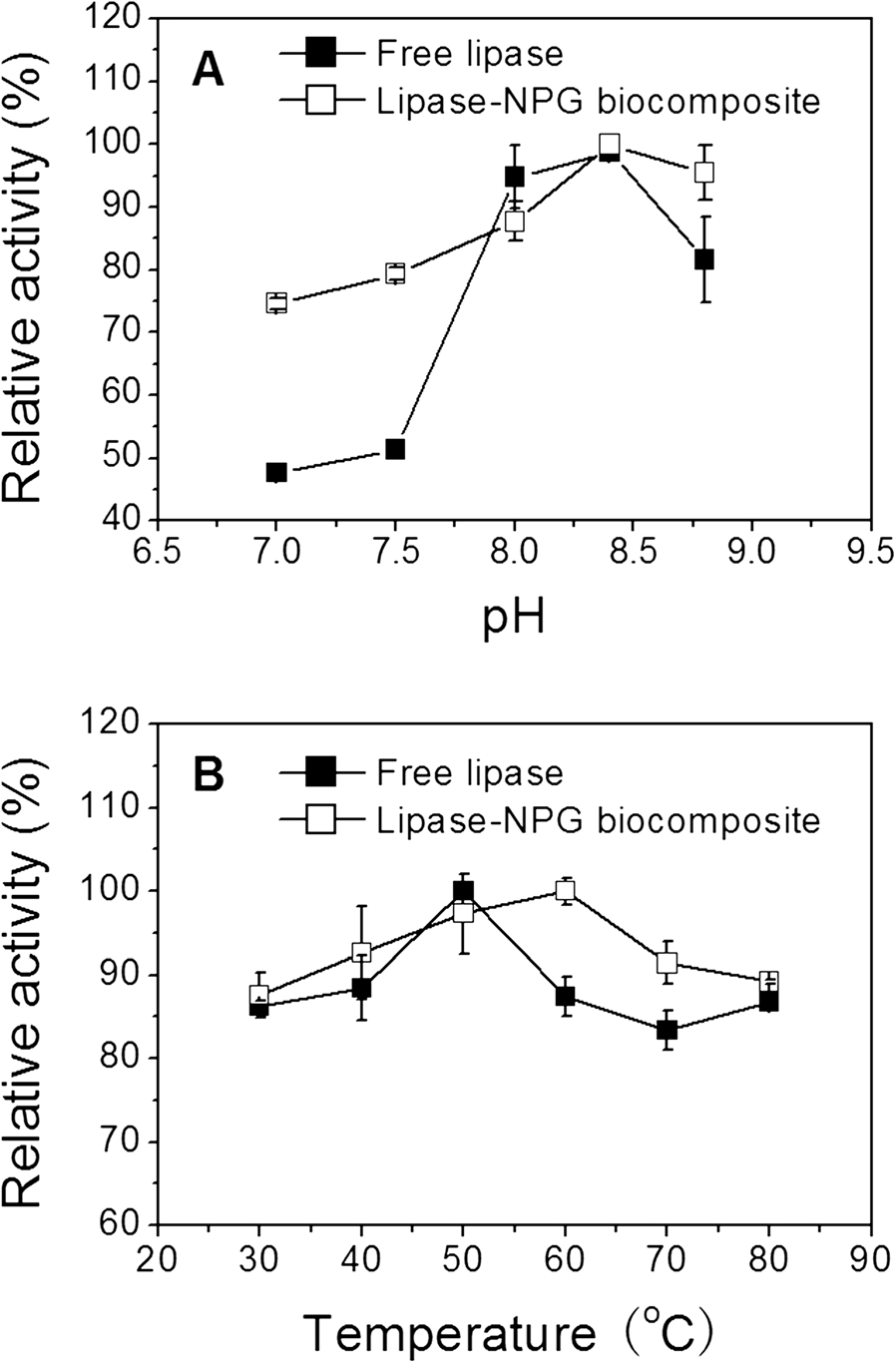
**Effect of buffer pH and temperature.** The effects of (**A**) pH and (**B**) temperature on the catalytic activities of free lipase and the lipase-NPG biocomposite with a pore size of 35 nm adsorbed for 72 h.

An enzyme in a solution may have a different optimal pH from that of the same enzyme immobilized on a solid matrix [24]. The catalytic activities of free lipase and the lipase-NPG biocomposites with a pore size of 35 nm were assayed at varying pH (7.0 to 9.0) at 40°C. The lipase-NPG biocomposite and free lipase had similar pH activity profiles with the same optimum activity at pH 8.4 (Figure 4A). Compared with free lipase, the lipase-NPG biocomposite maintained higher catalytic activity at a broader pH range, which could possibly offer a broader range of applications.

The effects of reaction temperature on the catalytic activity of free lipase and the lipase-NPG biocomposite with a pore size of 35 nm were also investigated by varying temperatures from 30°C to 80°C. Figure 4B shows that the maximum catalytic activity of the lipase-NPG biocomposite was observed at 60°C, whereas free lipase exhibited the highest activity at 50°C. Additionally, higher catalytic activity was maintained by the lipase-NPG biocomposite within the temperature range investigated. The improvement in the denaturation resistance of the lipase-NPG biocomposite was probably a consequence of increasing conformational stability by being adsorbed within nanoscale pore channels [24].

### Leaching test

**Figure 5:**
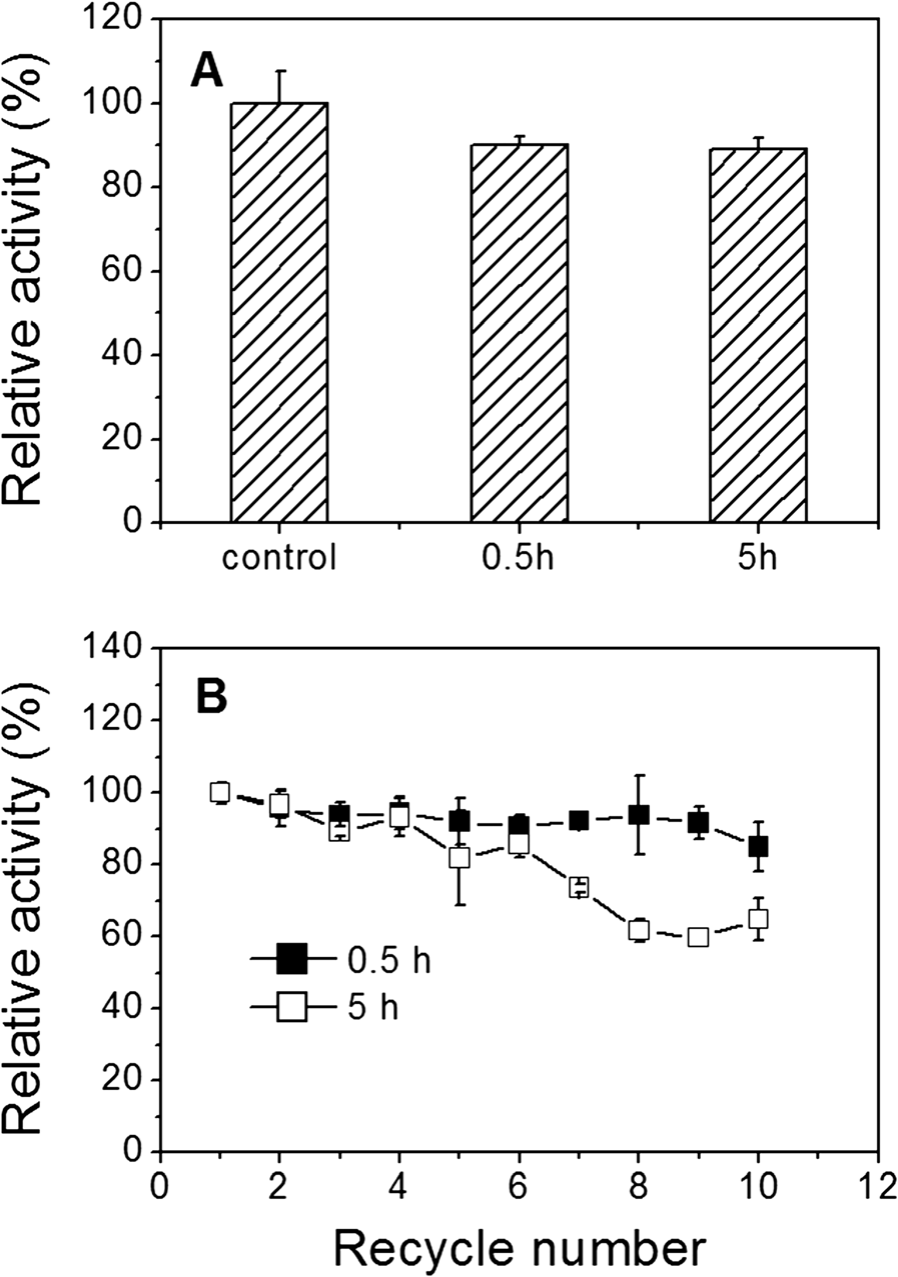
**Catalytic activity and reusability.** (**A**) Catalytic activity and (**B**) reusability after leaching test of the lipase-NPG biocomposite with a pore size of 35 nm adsorbed for 72 h.

Leaching has been one of the critical problems when porous materials were used as a support for the immobilization of enzymes, which could result in poor operational stability [6]. Therefore, the leaching of lipase from NPG was evaluated. Figure 5A shows that the lipase-NPG biocomposite with a pore size of 35 nm retained 90% and 89% of the initial catalytic activity after incubation for 0.5 and 5 h at 40°C, respectively. After incubation for 0.5 h, the reusability of the lipase-NPG biocomposite has no significant decrease, with 85% of the catalytic activity maintained after ten recycles (Figure 5B). After incubation for 5 h, the catalytic activity of the lipase-NPG biocomposite still retained 65% of the catalytic activity after ten recycles (Figure 5B). These results indicate that the leaching of lipase from NPG could be prevented by matching the protein’s diameter with pore size, which is consistent with the previous report that mesoporous silica with a pore size of 15 to 20 nm comparable to the dimensions of aldolase antibody 84G3 (hydrodynamic radius 8 nm) was specially prepared to enhance the immobilized enzyme stability and activity [25]. In contrast, approximately 50% loss in activity of lipase (average molecular diameter 5 nm) immobilized on mesoporous silica with a larger pore size of 62 nm was observed after 8 cycles, which attributed to leaching during the reaction and recovery of the immobilized enzyme [26].

## Conclusions

In conclusion, NPG with a three-dimensional spongy morphology was demonstrated to be a suitable support for lipase immobilization. The pore size of NPG and adsorption time played key roles in achieving high stability and reusability. The resulting lipase-NPG biocomposites with a pore size of 35 nm exhibited excellent catalytic activity and stability compared with the native lipase at different pH and temperatures. The leaching of lipase from NPG could be prevented by matching the protein’s diameter and pore size. These results suggest that NPG with unique structure properties has great potential for applications in biomolecule separation systems, biocatalysis, electrocatalysis, and biosensors.

## Competing interests

The authors declare that they have no competing interests.

## Authors’ contributions

XD and XL designed the experiments and carried out the characterization. YL and CW participated in the NPG and lipase-NPG biocomposite fabrication. XW and PX made substantial contributions to the conception and design of this paper. XW and XD wrote the paper. All authors read and approved the final manuscript.
